# Molecular epidemiology of SARS-CoV-2 clusters caused by asymptomatic cases in Anhui Province, China

**DOI:** 10.1186/s12879-020-05612-4

**Published:** 2020-12-07

**Authors:** Yuan Yuan, Jun He, Lei Gong, Weiwei Li, Liangliang Jiang, Jiang Liu, Qingqing Chen, Junling Yu, Sai Hou, Yonglin Shi, Siqi Lu, Zhuhui Zhang, Yinglu Ge, Nan Sa, Lan He, Jiabing Wu, Yong Sun, Zhirong Liu

**Affiliations:** 1 Anhui Provincial Center for Disease Control and Prevention, 12560, Fanhua Avenue, Hefei, China; 2 Key Laboratory for Medical and Health of the 13th Five-Year Plan, 12560, Fanhua Avenue, Hefei, Anhui China; 3 Maanshan Center for Disease Control and Prevention, 849, Jiangdong Avenue, Maanshan, China; 4 Huainan Center for Disease Control and Prevention, Linchang Avenue, Huainan, China

**Keywords:** Coronavirus, SARS-CoV-2, Cluster, Asymptomatic case, Spike gene

## Abstract

**Background:**

COVID-19 is a newly emerging disease caused by a novel coronavirus (SARS-CoV-2), which spread globally in early 2020. Asymptomatic carriers of the virus contribute to the propagation of this disease, and the existence of asymptomatic infection has caused widespread fear and concern in the control of this pandemic.

**Methods:**

In this study, we investigated the origin and transmission route of SARS-CoV-2 in Anhui’s two clusters, analyzed the role and infectiousness of asymptomatic patients in disease transmission, and characterized the complete spike gene sequences in the Anhui strains.

**Results:**

We conducted an epidemiological investigation of two clusters caused by asymptomatic infections sequenced the spike gene of viruses isolated from 12 patients. All cases of the two clusters we investigated had clear contact histories, both from Wuhan, Hubei province. The viruses isolated from two outbreaks in Anhui were found to show a genetically close link to the virus from Wuhan. In addition, new single nucleotide variations were discovered in the spike gene.

**Conclusions:**

Both clusters may have resulted from close contact and droplet-spreading and asymptomatic infections were identified as the initial cause. We also analyzed the infectiousness of asymptomatic cases and the challenges to the current epidemic to provided information for the development of control strategies.

## Background

The emergence of new human viral diseases affecting the respiratory tract continues to threaten global public health. Subsequent virological testing revealed a novel CoV in these patients [1]. This new human coronavirus (CoV) strain (SARS-CoV-2) was named by the International Committee on Taxonomy of Viruses (ICTV), and pneumonia resulting from infection with this coronavirus was named “COVID-19” [2]. Coronaviruses, which are widely found in humans and other mammals as well as avian species, cause asymptomatic infections or respiratory tract disorders. The SARS-CoV-2 virus is a new genus Coronavirus of the family Coronaviridae, which contains four other genera, Alphacoronavirus, Betacoronavirus, Gammacoronavirus, and Deltacoronavirus, based on phylogenetics and serology [3]. Among the Coronaviridae that cause human illness, NL63, HKU1, 229E, and OC43, cause a mild infection often resembling the common cold [4, 5], whereas the severe acute respiratory syndrome coronavirus (SARS-CoV) and Middle East respiratory syndrome coronavirus (MERS-CoV) cause acute respiratory distress syndrome (ARDS) and are associated with high mortality rates [6]. SARS-CoV-2 was found to be a member of the beta coronavirus family, and the same species as SARS-CoV and SARS-related bat CoVs [7]. Patterns of the spread of infection indicate that SARS-CoV-2 can be transmitted by respiratory droplets and physical contact, and might be more transmissible than SARS-CoV [8, 9]. On January 30, 2020, the World Health Organization (WHO) declared the virus an international public health emergency. There has been an alarming increase in the number of cases worldwide and several reports indicate asymptomatic transmission [10–12]. The contribution of asymptomatic cases with SARS-CoV-2 to the transmission is not well characterized, but may play a role in transmission, thus, posing a major challenge to infection control.

The CoV spike (S) protein that protrudes from the surface of virions is the major antigenic protein. Spike proteins are often functionally divided into two subunits, S1 and S2. S1 contains the receptor binding domain (RBD), while S2 is responsible for fusion with the cellular membrane [13, 14]. The S1 subunit is composed of two distinct domains, the N-terminal domain (NTD) and the C-terminal domain (CTD), both of which interact directly with host receptors [13]. Compared with the open reading frame (ORF)1a and ORF1b sequences in coronaviruses, the spike protein often has the most variable amino acid mutations. Characterization of SARS-CoV-2 spike proteins indicates that this structure binds the same receptor (angiotensin-converting enzyme 2; ACE2) as SARS-CoV, which is expressed in both the upper and lower human respiratory tracts [15]. It was recently reported that the ectodomain of the SARS-CoV-2 spike protein binds to the PD of ACE2 with a Kd of approximately 15 nM [16]. SARS-CoV-2 RBD has a stronger interaction with ACE2, which is likely due to the major role of a unique phenylalanine F486 in the flexible loop of the spike protein [17].

To gain a better understanding of the origin and transmission patterns of asymptomatic SARS-CoV-2 carriers in this study, we used virus sequencing as the basis of a molecular epidemiological analysis of two clusters caused by asymptomatic cases in Anhui Province, China. The clinical, virological and epidemiological features of the infections showed that asymptomatic cases have the potential to excrete viruses and become a source of infection. Social distancing and the correct perception of the threat to public health are important factors in controlling the spread of the disease. Our nucleotide sequence alignment also revealed 16 mutations contained within the entire spike gene. Mutations in the spike glycoprotein may induce conformational changes, which probably lead to changes in antigenicity. Therefore, the identification of these mutated amino acids is of great significance and warrant further investigation.

## Methods

### Patients and samples

In terms of epidemiology, a cluster is defined by the Novel Coronavirus Pneumonia Prevention and Control Program (6th trial version) as two or more confirmed cases or asymptomatic infections found in a small area (such as home, office, work unit, school class workshop, etc.) within 14 days, with a possibility of interpersonal transmission due to close contact or infection due to co-exposure. An asymptomatic case is defined as an individual without clinical symptoms but who tests positive by SARS-2-specific RT-PCR of respiratory tract and other specimens or is serum SARS-2-specific IgM positive. Asymptomatic cases are identified mainly through close contact screening, clustered case investigation and source tracking investigation. According to the Practice Guidance for Diagnosis and Treatment of Novel Coronavirus Pneumonia in China (7th trial version) published by the China National Health Commission, a patient with COVID-19 is diagnosed based on epidemiological history and clinical symptoms, and is confirmed by one of the following methods: real-time reverse transcription polymerase chain reaction (RT-PCR) assay, high-throughput genome sequencing, or serological measurements of anti-viral immunoglobulin M (IgM) and G (IgG) antibodies. The asymptomatic cases reported in this study were identified through contact tracing. A standardized surveillance reporting form was used to collect epidemiologic and clinical data, including demographic characteristics, recent exposure history, and clinical signs and symptoms.

### RNA extraction, RT-PCR and gene sequencing

RNA was extracted from throat swabs and sputum samples using a QIAGEN OneStep RT-PCR Kit (Qiagen, Germany), according to the manufacturer’s instructions. Specific real-time reverse transcription polymerase chain reaction (rRT-PCR) assays were performed to identify SARS-CoV-2 virus. The nucleotide sequence of the whole spike gene was obtained by direct sequencing. Briefly, the extracted RNA was amplified with five pairs of primers using the OneStep RT-PCR System (QIAGEN GmbH, GERMANY) with Hot StarTaq DNA polymerase (Veriti96-well Thermal Cycler, Applied Biosystems, USA). The sequences of the primers used in this study are listed in Table 1. The PCR products were purified with a Wizard DNA Clean-Up System (A7280, Promega, USA). The purified PCR products were then sequenced using BigDye Terminator v3.1 Cycle Sequencing Kit (Applied Biosystems) and analyzed with an ABI 3730XL DNA Analyzer (Applied Biosystems). Spike gene sequences were deposited in the NCBI database.

**Table 1:** Spike gene and reference primer pairs used in this study

Primer name	Primer sequence(5′ → 3′)	Primer location	Primer reference
AF^a^	5′-CGC GAA CAA ATA GAT GGT TA-3’	21,320–21,339	MN908947.3
AR^b^	5′-CTG CAG CAC CAG CTG TCC AA-3’	22,368–22,387	MN908947.3
BF	5′-AGG GAA TTT GTG TTT AAG A-3’	22,146–22,164	MN908947.3
BR	5′-AAC ACC TGT GCC TGT TAA A-3’	23,231–23,249	MN908947.3
CF	5′-CCG GTA GCA CAC CTT GTA A-3’	23,002–23,020	MN908947.3
CR	5′-AAT GAG GTC TCT AGC AGC A-3’	24,128–24,146	MN908947.3
DF	5′-CCA GAT CCA TCA AAA CCA A-3’	23,997–24,015	MN908947.3
DR	5′-CTG GTG ATG TAT GAT TCT T-3’	25,065–25,083	MN908947.3
EF	5′-CTG CTC CTG CCA TTT GTC A-3’	24,808–24,826	MN908947.3
ER	5′-TTG GAG AGT GCT AGT TGC C-3’	25,632–25,650	MN908947.3

^a^F, Forward primer

^b^R, Reverse primer

### Phylogenetic analysis

Reference spike gene sequences were downloaded from the NCBI nucleotide sequence database (http://www.ncbi.nlm.nih.gov). The bat coronavirus RaTG13 and pangolin (Guangdong1, GuangxiP4L) coronavirus sequences were downloaded from GISAID (http://www.GISAID.org). Spike sequences were aligned with reference sequences using MAFFT software (version 7.450). Phylogenetic analyses of the complete spike gene and major coding regions were conducted using RAxML software (version 8.2.9). Using the model of GTR + G nucleotide substitution in RAxML software, the maximum likelihood genetic evolutionary tree was constructed by 1000 bootstrap tests [18]

## Results

### Demographic characteristics of cases

**Fig. 1:**
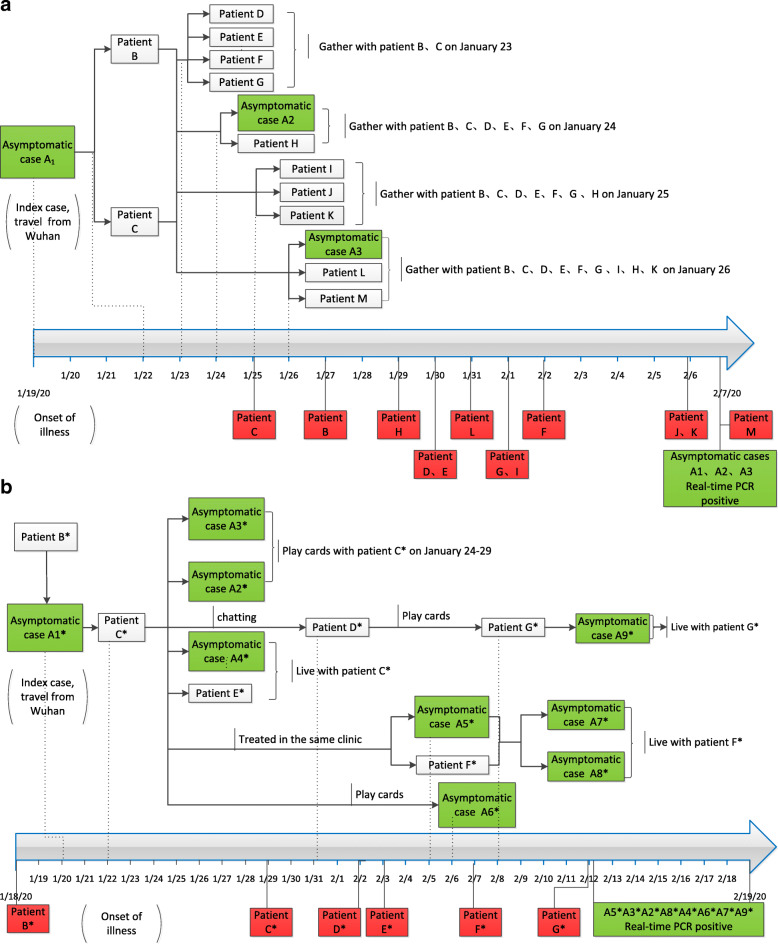
**a** (Cluster1), **b** (Cluster 2). Schematic representation of the relationships between the patients and the asymptomatic cases. Chronology of symptom onset and identification of positive SARS-CoV-2 findings among the family cluster. The gray dotted line indicates the time of the initial exposure of the cases and the solid line indicates the time of onset of the illness and the date of positive examination in asymptomatic cases; the asymptomatic cases are marked with a green box and the patient is marked with a red box after disease onset

The first cluster outbreaks caused by asymptomatic cases comprised 12 confirmed cases (B–M) and three asymptomatic cases (A1–A3). Patient C first developed a fever, cough and muscle soreness on January 25, 2020, and the last case, M, developed a fever on February 7, 2020. The epidemic occurred in three generations of 15 cases. An asymptomatic case, A1, was used as the index patient, while the second generation comprised patients B and C, and the third generation comprised asymptomatic cases A2, A3, and patients D–M. Three patients (Patients B, L and M) were single exposure cases, while the rest were multiple exposure cases. According to the analysis of the onset time in single exposure cases, the incubation period ranged from 4 to 11 days (Patient B, 4 days; Patient L, 4 days, and Patient M, 11 days; Fig. 1a).

The second cluster outbreaks caused by asymptomatic case consisted of six confirmed cases, Patients B*–G*and nine asymptomatic cases, A1*–A9*. Patient B* first developed fever and weakness on 18 January, 2020, and the last case, G*, developed a cough on 12 February, 2020. Four generations of cases of transmission occurred in this outbreak. Patient B* and asymptomatic case A1* were the first generation and the second-generation was patient C*. In the third generation, there were five asymptomatic cases, A2*–A6*, and three patients D*–F*. The fourth generation comprised patient G* and three asymptomatic cases, A7*–A9*. Patient C*and D* were single exposure; the incubation periods of patients C* and D* were 6 days and 1 day, respectively (Fig. 1b) A summary of clinical features and microbiological results from clinical specimens collected from the two clusters of patients infected with SARS-CoV-2 at presentation are shown in Table 2.

**Table 2:** Summary of clinical features and microbiological results from clinical specimens collected from the two clusters infected with SARS-CoV-2 at presentation

**Cluster 1**															
	Case A1	Patient B	Patient C	Patient D	Patient E	Patient F	Patient G	Patient H	Case A2	Patient I	Patient J	Patient K	Patient L	Patient M	Case A3
Relationship	Friend of patient C	Wife of patient C	Husband of patient C	Sun of patients B and C	Relative of patient B and C	Relative of patient B and C	Relative of patient B and C	Relative of patient B and C	Relative of patient B and C	Relative of patient B and C	Relative of patient B and C	Relative of patient B and C	Relative of patient B and C	Relative of patient B and C	Relative of patient B and C
Occupation	Merchant	Worker	Driver	Student	Farmer	Student	Worker	Worker	Student	Farmer	Farmer	Farmer	Worker	Farmer	Farmer
Days since onset	/	5	3	7	7	10	9	5	/	6	12	12	5	12	/
Presenting symptoms and signs	··	··	··	··	··	··	··	··	··	··	··	··	··	··	··
Fever (Body temperature)	–	+	+(37.3 °C)	+(39 °C)	+(37.5 °C)	–	+	+	–	–	–	–	+	+	–
Cough	–	+(productive)	–	+	+(dry)	–	–	–	–	–	+(dry)	+(dry)	+(productive)	–	–
Generalised weakness	–	–	–	–	+	–	–	–	–	–	–	–	+	–	–
Sneezing	–	–	–	–	–	–	–	–	–	+	–	–	–	–	–
Sore throat	–	–	–	–	–	+	–	–	–	–	–	–	–	–	–
Diarrhoea	–	–	–	–	–	–	–	–	–	–	–	–	–	–	–
Muscle soreness	–	+	–	+	–	–	–	–	–	–	–	–	–	–	–
Vomitting	–	–	–	–	–	–	–	–	–	–	–	–	+	–	–
backpain	–	–	–	–	–	–	–	–	–	–	–	–	–	–	–
Pneumonia	–	–	–	–	–	–	–	–	–	–	–	–	–	–	–
Sample information	··	··	··	··	··	··	··	··	··	··	··	··	··	··	··
Sample type	Throat swab	sputum	Throat swab	Throat swab	Throat swab	Throat swab	Throat swab	Throat swab	Throat swab	Throat swab	Throat swab	Throat swab	sputum	Throat swab	Throat swab
Strains	HN022	HN012(MT415367)	HN021	HN011(MT415366)	HN017(MT415369)	HN025	HN026	HN020(MT415370)	HN018	HN019	HN015(MT415368)	HN023(MT415371)	HN016	HN027	HN024(MT415372)
Real-time RT-PCR (N gene) Ct value	36.22	28.88	35.2	26.86	27.55	33.68	32.5	25.5	30.76	32.7	27.66	28.69	36.76	33.25	26.2
Spike Genome sequence obtained	Partial	Complete	ND	Complete	Complete	ND	ND	Complete	ND	ND	Complete	Complete	ND	ND	Complete
Percent similarity to Wuhan-HB01(S gene)	/	99.9	/	99.9	100	/	/	99.9	/	/	100	99.9	/	/	100
**Cluster 2**															
	Patient B*	Patient C*	Patient D*	Patient E*	Patient F*	Patient G*	Case A1*	Case A2*	Case A3*	Case A4*	Case A5*	Case A6*	Case A7*	Case A8*	Case A9*
Relationship	Husband of case A1*	Friend of case A1*	Neighbor of patient C*	Daughter-in-law of patient C*	Live in the same village with patient C*	Friend of patient D*	Wife of Patient B*	Neighbor of patient C*	Neighbor of patient C*	Mother of patient C*	Husband of patient F*	Neighbor of patient C*	Sun of patient F* and case A5*	Mother of case A5*	Wife of Patient G*
Occupation	Merchant	Farmer	Farmer	Merchant	Merchant	Farmer	Merchant	Merchant	Merchant	Farmer	Merchant	Farmer	/	Farmer	Farmer
Days since onset	0	7	2	12	2	4	/	/	/	/	/	/	/	/	/
Presenting symptoms and signs	··	··	··	··	··	··	··	··	··	··	··	··	··	··	··
Fever (Body temperature)	+(38 °C)	+	–	+(37.9 °C)	–	+(38 °C)	–	–	–	–	–	–	–	–	–
Cough	–	+(productive)	+(dry)	–	–	+(productive)	–	–	–	–	–	–	–	–	–
Generalised weakness	+	–	–	–	+	–	–	–	–	–	–	–	–	–	–
Sneezing	–	–	–	–	–	–	–	–	–	–	–	–	–	–	–
Sore throat	–	–	–	–	–	–	–	–	–	–	–	–	–	–	–
Diarrhoea	–	–	–	–	–	–	–	–	–	–	–	–	–	–	–
Muscle soreness	–	–	–	–	–	–	–	–	–	–	–	–	–	–	–
backpain	–	+	–	–	–	–	–	–	–	–	–	–	–	–	–
Pneumonia	+	+	+	+	+	+	–	–	–	–	–	+	–	–	+
Sample information	··	··	··	··	··	··	··	··	··	··	··	··	··	··	··
Sample type	Throat swab	sputum	Throat swab	Throat swab	Throat swab	sputum	Throat swab	Throat swab	Throat swab	Throat swab	Throat swab	Throat swab	Throat swab	Throat swab	Throat swab
Strains	MAS005(MT415373)	MAS326(MT415374)	MAS425	MAS469	MAS328	MAS581	MAS633	MAS390	MAS368(MT415375)	MAS596	MAS332	MAS656	MAS661	MAS525(MT415376)	MAS635(MT415377)
Real-time RT-PCR (N gene) Ct value	29.15	28.21	25.37	34.65	34.31	24.57	–	33.45	26.63	34.65	20.31	31.22	33.57	22.96	26.29
Spike Genome sequence obtained	Complete	Complete	ND	ND	ND	ND	ND	ND	Complete	ND	ND	ND	ND	Complete	Complete
Percent similarity to Wuhan-HB01 (S gene)	99.9	99.9	/	/	/	/	/	/	100	/	/	/	/	100	99.9

+ = positive, – = negative, *ND* not detected. Case A1-A3 = Asymptomatic case A1-A3, Case A1*-A9* = Asymptomatic case A1*-A9*

### Genetic characterization of the virus

The rRT-PCR results of the original clinical samples obtained from the 30 patients in this study are shown in Table 2. Twelve complete SARS-CoV-2 spike gene sequences were obtained; these data have been deposited in the NCBI database (accession number: MT415366–MT415377).

We constructed phylogenetic trees based on the nucleotide sequences of these spike gene. The twelve complete spike gene sequences were nearly identical across the whole spike, with sequence identity exceeding 99.9, and 99.8%–100% similarity between the Anhui and Wuhan strains. We then performed phylogenetic analysis of the collection of coronavirus sequences from NCBI and GS. The phylogenetic tree showed that 12 complete spike gene sequences clustered with seven other spike sequences of viruses belonging to the Betacoronavirus genera from different countries and regions. In terms of phylogeny, RaTG13, Pangolin-CoV and SARS-CoV-2 were clustered into a well-supported group. SARS-CoV-2 and RaTG13 were grouped together within this group, with Pangolin-CoV as their closest common ancestor.

The spike gene cluster was situated with the groups of SARS coronaviruses, and its inner joint neighbors were bat coronavirus RaTG13 or pangolin coronaviruses, with human bat coronavirus and MERS coronaviruses as the outgroup (Fig. 1a). Compared to the spike gene of SARS-CoV and MERS-CoV, the SARS-CoV-2 strains were less genetically similar to SARS-CoV (79.8–80.3% similarity) and MERS-CoV (53.7% similarity). The similarity plot suggested that RaTG13 was the most closely related sequence to SARS-CoV-2 throughout the spike sequences. SARS-CoV-2 and RaTG13 showed 94.6–94.7% sequence similarity. The strains in this study were similar to the pangolin Guangdong strain and the Guangxi strain (82.6–86.9% similarity) .

**Fig. 2:**
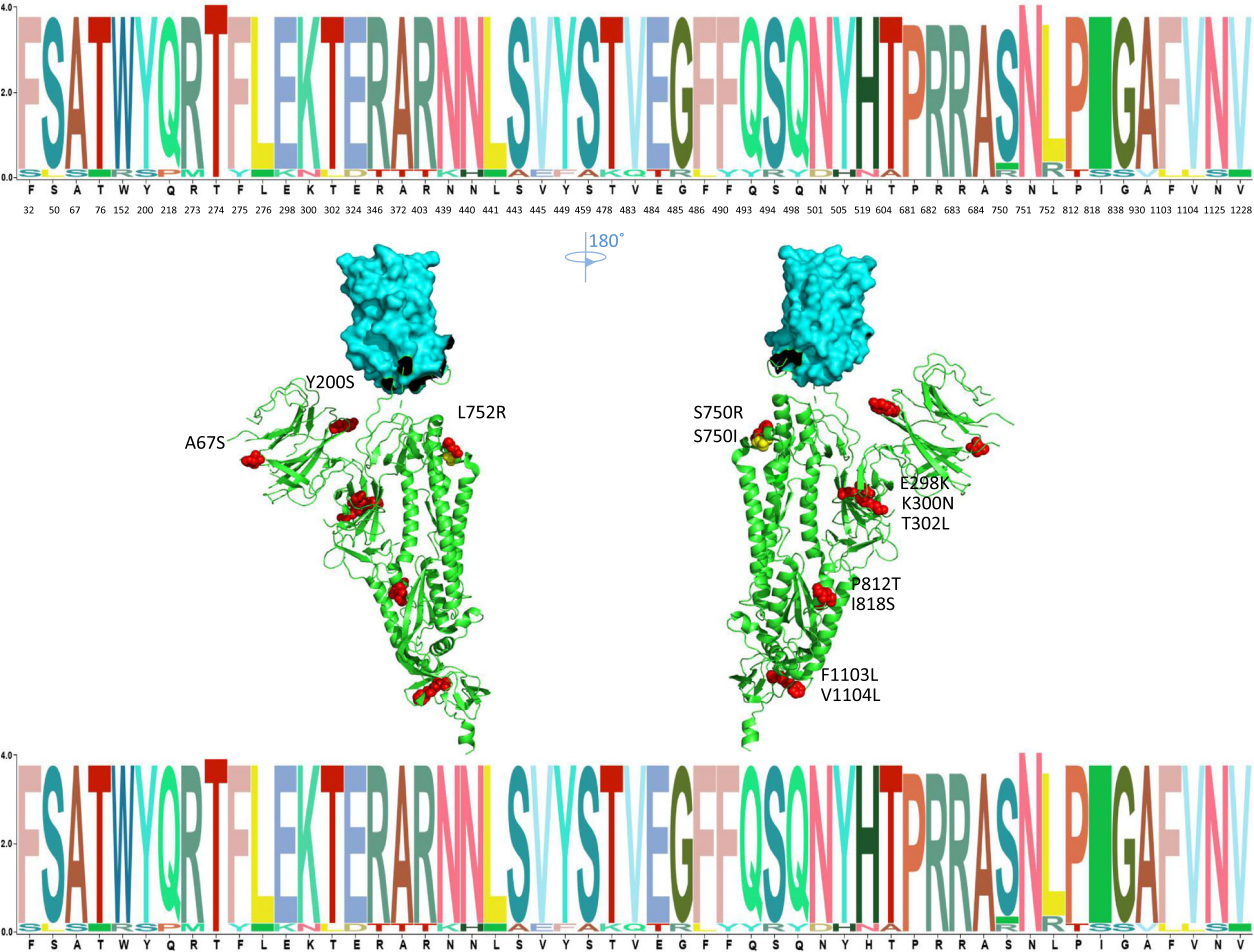
Predicted 3D structures of spike proteins of SARS-CoV-2. Graphical representation of multiple SARS-CoV-2 spike protein amino acid sequence alignment (Wuhan-Hu-1, RaTG13 and Anhui strains). One stack for each position in the sequence. Numbers are based on the Wuhan-Hu-1 sequence. The overall height of the stack indicates the sequence conservation at that position, while the height of symbols within the stack indicates the relative frequency of each amino acid at that position. The model was built on the basis of the structure of SARS-CoV-2 spike glycoprotein (Protein Data Bank ID: 6vsb.1.A). Spike proteins were aligned using PyMOL. Blue is the 335-516RBD region of the spike protein is shown in blue and the red circle highlights the presence of a variable region in the spike proteins of Anhui strains. Due to the lack of 152, 838 and 485 sites in the model, the three variations W152R, G838S and G485R are not marked

Sequencing of throat swab and sputum samples collected after the onset of the illness revealed 16 single nucleotide variations within the spike gene (Table 3). The protein structures were predicted based on spike proteins (PDB_ID:6VSB) in the National Genomics Data Center database (https://bigd.big.ac.cn/) using PyMOL. We evaluated amino acid variations in the spike proteins among the Sarbecovirus coronaviruses. Five variations of these sequences occurred in only one isolate. In addition, 1–3 single nucleotide variations were identified in seven samples. In the first cluster, amino acid mutations were detected in the following strains: HN011: E298K, K300N, T302L, L752R, and P812T; HN015: G485R, HN020: A67S, and F1103L; HN023: Y200S, I818S, and V1010L. In the second cluster, amino acid mutations were detected in the following strains: MAS005: S750R, and L752R; M368: G838S; and M525: W152R; M635: S750I. Most of these mutations were located in non-RBD regions, except for one sample, HN015, in which a G485R mutation was detected in the RBD (Fig. 2).

**Table 3:**
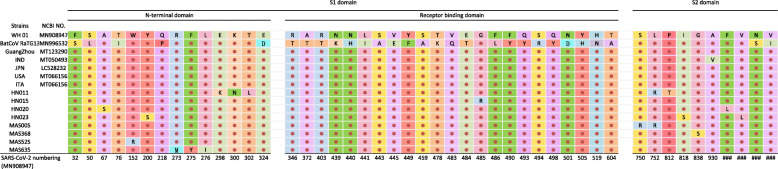
Specific amino acid variations among the spike proteins of the SARS-CoV-2 and the bat coronaviruses. One-letter codes represent amino acids. CoV = coronavirus.

### Epidemiological investigation of the two sources of the cluster

In the epidemiological traceability investigation of the first cluster comprising patients B and C, it was found that asymptomatic case A1 had eaten a meal with patient B and C in an enclosed space (10 m^2^) on January 22, 2020. When asymptomatic case A1 was questioned initially, he deliberately concealed his history, and only admitted traveling from Wuhan after tracking information was obtained from public security authorities. The same pattern was identified in the second cluster, in which asymptomatic case A1* admitted that she and her husband had traveled from Wuhan only after several public security investigations.

In the first clustered case, the second-generation case patient B had not visited Wuhan during the 14 days before the onset of symptoms. On January 27, 2020, she developed a productive cough, fever and muscle soreness. On February 2, 2020, she went to the local hospital for treatment. The viral nucleic acid test was positive on February 3, and she was confirmed as COVID-19 on February 4.

The mode of transmission in the first cluster was close contact and droplet-spreading in a family gathering. After the asymptomatic case A1 returned from Wuhan on January 19, and had meals with patients B and C, it was inferred that asymptomatic case A1 was the source of infection of patients B and C; the rest of the cases were relatives of patients B and C. This cluster outbreak was caused by close contact during multiple meals within a family setting.

In the second cluster case, asymptomatic case A1* and her husband patient B* began selling tofu in a vegetable market in Wuhan from November 2019. Patient B* developed a fever and weakness on January 18, 2020 and was treated in a small local clinic. The couple traveled from Wuhan on January 20. On January 22, asymptomatic case A1* went to a banquet at the same time her husband, patient B*; he was reported to have traveled from Wuhan by his neighbor and had a fever. Patient B* was immediately sent to a local hospital for isolation and treatment and was diagnosed as COVID-19 on 24 January.

Patient C* owned a snack business with her husband in Zhejiang Province. She and her husband returned to Anhui with two neighbors on January 18. Patient C* attended a banquet on January 22 and played cards with the asymptomatic case A1*. She developed a fever, productive cough, and backpain on January 29 and went to the local hospital on February 1. Her chest CT showed pneumonia in both lungs and she tested positive for viral nucleic acids by rRT-PCR on February 9 when she was diagnosed as COVID-19. Prior to the onset illness, patient C* lived with her daughter-in-law, patient E*, and her mother, asymptomatic case A4*. Patient E* developed a fever on February 3. Throat swabs collected from her mother A4* on February 17 tested positive by rRT-PCR, although she did not have any symptoms such as fever or discomfort. Patient F* was accompanied by her husband A5* and treated by fluid infusion in a small clinic on February 5–6, together with patient C*. She had symptoms of discomfort on February 7 and tested positive by rRT-PCR on February 11. She lived with her husband patient A5*, mother-in-law patient A8*, and her 4-year-old son patient A7*, but all were asymptomatic cases.

The main mode of transmission in the second cluster was social activities - playing cards, social gatherings and close contact. In the early phase of the outbreaks, the population had no perception of the risk of the disease.

## Discussion

The unprecedented SARS-CoV-2 infection has been a global public health concern since it was first identified in December, 2019. The most common topic of concern is the possibility of widespread virus transmission by asymptomatic carriers. Here, we report two clusters of COVID-19 in the first wave epidemic in Anhui Province, China. In the first cluster, a couple (case B and case C) was infected with SARS-CoV-2 during a meal in an enclosed space with asymptomatic case A1 (index case). The throat swab collected from asymptomatic case A1 was found to be viral nucleic acid positive, 19 days after his return from Wuhan, although complete spike genes were not amplified from subsequent samples. We speculate that asymptomatic case A1 may have produced virus-specific antibodies by this time. The rRT-PCR test of the throat swab sample confirmed only the presence of virus nucleic acid fragments in this patient and no live virus was isolated from the sample.

Similarly, in the second clustered case, patient C* was diagnosed first. Epidemiological investigations revealed that she had close contact with asymptomatic case A*, although she had no direct contact with patient B*. However, the spike gene sequences of the two strains from patients B*and C* showed almost complete nucleotide identity and a synonymous single nucleotide mutation C2461T was identified, suggesting that the most likely scenario is that the virologically documented patient with pneumonia (patient C*) acquired the infection from asymptomatic case A*. The systemic case A1* was identified during an epidemiological survey of close contacts of patient C* on 10 February. It had been 21 days since she had returned from Wuhan and her throat swab tested negative by rRT-PCR; however, on February 18, serological tests of asymptomatic case A1* revealed that she was positive for virus-specific IgM antibodies. The clinical manifestations of COVID-19 patients have been reported to range from asymptomatic cases to mild and severe symptoms. Laboratory tests of viral nucleic acids can yield false-negative results, and serological testing of virus-specific IgG and IgM should be used as an alternative for diagnosis [19]. It has been reported that the viral load peaked during the first week of the illness then gradually declined over the second week [20]. We also found that after SARS-CoV-2 was passed from the second-generation cases to the third generation cases, the family of the third generation patient F* were all asymptomatic cases, including her husband A5*, her four-year-old son A7* and her mother-in-law A8* (A7 *and A8* were fourth generation cases). Notably, the throat swab was collected from asymptomatic A8* on February 16, which might be closer to the date of her infection. The virus load of this sample was high, and the virus was also isolated cultured in VERO cells. The high viral load during the early phase of the illness suggests that patients might be most infectious during this period, and might also account for the high transmissibility of SARS-CoV-2 [21]. Failure to detect of the virus in patient A8* in the early stages of the infection might have increased the risk of rapid spread of the disease in the crowded conditions. In these two clustered outbreaks caused by asymptomatic cases, both had epidemiological evidence suggesting that infection was acquired directly from Wuhan, and also that a few index cases might have caused a disproportionately high number of secondary cases. In these two clusters, asymptomatic cases were found in retrospective surveys and serological investigations of close contact populations after the outbreak. Because asymptomatic cases have no obvious clinical symptoms, it is difficult to detect them during the incubation period or initial phase without active detection. As a source of infection, these asymptomatic cases can cause outbreaks, which are extremely harmful to society. At present, China has basically controlled the domestic epidemic situation by implementing measures of social distancing, avoidance of large gatherings and strengthening of the detection and screening of the resident and inbound populations. Analysis of the existing cases reported in China has shown significant increases in the number and proportion of asymptomatic infections, indicating that our prevention and control measures are effective in capturing people in the initial phase of infection. Furthermore, these findings reflect the importance of timely detection of the source of asymptomatic infections to prevent the rebound and provide scientific data as a reference for the worldwide fight against SARS-CoV-2.

In our phylogenetic analysis of SARS-CoV-2, we sequenced samples from 12 patients and showed that the virus belongs to the subgenus Sarbecovirus. The spike gene analysis revealed that SARS-CoV-2 showed higher level of similarity with a bat-derived coronavirus strain TG13 than with the virus that caused the SARS outbreak in 2003. Epidemiologically, in the two clusters of cases we studied, both the index cases had returned from Wuhan, showing that these individuals may have had close contact with the source of infection in Wuhan. During the travel rush in the peak of the Chinese Spring Festival, the virus was spread to friends, neighbors and family. The spike gene sequences of the SARS-CoV-2 isolated from the patient samples in these two epidemic outbreaks were almost identical (99.8–100% similarity). This finding indicates that although the outbreaks occurred in different areas of Anhui Province, the sources may have been identical. As the virus spreads quickly in a short period of time, mutations must be continuously monitored.

As a typical RNA virus, coronaviruses evolve at an average rate of approximately 10^− 4^ nucleotide substitutions per site per year, mutating in each replication cycle [22]. During evolution, high-frequency homologous RNA recombination and gene mutations are thought to be the main forces driving coronavirus adaptation to specific hosts, leading to the emergence or outbreaks of new strains or genotypes [23, 24]. Amino acid substitutions in the surface proteins produce antigenic variation that helps the virus to evade the host immune system. This phenomenon is a major driving force for viral evolution and an important adaptation strategy that enhances the persistence of a virus by evading host immune pressure [25, 26]. To date, a total of 72 mutation sites in the spike protein of SARS-CoV-2 have been reported, including 14 amino acid mutations in two or more strains of the virus. Two mutations, V483A and V367A, have been identified in the locus responsible for the binding of ACE2 had (https://bigd.big.ac.cn/ncov/protein). There are 14 amino acid residues in the SARS-CoV RBD that are in direct contact with ACE2; eight of these positions are strictly conserved and the other six are (semi)conservatively conserved (including Asn439, Asn501, Gln493, Gly485 and Phe486; SARS-CoV-2 number) [7, 17]. Our research showed amino acids 335–516 of the RBD of spike are highly conserved, and only one strain showed a change (G485R) in the variable region. This is undoubtedly a good news, indicating that the virus has undergone normal mutations during the process of replication only after multiple passages in the population and that this region represents a potential target for development of vaccines against the SARS-CoV-2.

The functions of the NTD and the reasons for its variability are not yet known. Studies have shown that, although the NTD is not essential for binding to hACE2, deletion of the NTD markedly reduced both binding and membrane fusion [27]. One possibility is that the NTDs may exhibit antigenic variation, participate in binding processes or subtle remodeling of antigenic evolution, providing a mechanism to evade neutralization by host immune responses. This mechanism is utilized by other respiratory viruses, such as influenza viruses [28, 29]. We found five spike protein sequences mutations in the NTD, among which the AA substitution seems to be a common evolutionary strategy for CoV, as high variability of NTDs has also been detected in BCoV, OC43 and HCoV-NL63 [30–32]. Therefore, investigation of the virus isolates with variations in the spike gene is crucial to elucidate the influence of such mutations on host receptor binding activity and to clarify the presumed difference in disease phenotype.

Limitations of the study should be noted. In the early phase of outbreak, the epidemic was serious, and medical workers were dedicated to treating COVID-19 patients. Samples from asymptomatic cases were rarely obtained, and some cases had incomplete files. Furthermore, in the early stage of the epidemic, medical workers focused mainly on follow-up investigations of patients, and initiatives to screen asymptomatic people were not implemented in a timely manner, resulting in delayed sampling of these two index cases. Finally, both of these clusters occurred during the most severe outbreak in Anhui Province, when there were insufficient human and material resources to continuously sample and observe the infectiousness of asymptomatic infections.

## Conclusions

In summary, the use of molecular epidemiology to complement conventional epidemiology provides additional understanding of the transmission of the disease. These data confirm that during the early phase of an epidemic, a combination of rigorous and timely epidemiological investigations and multiple detection methods could help to identify asymptomatic individuals, which will have a major effect on controlling the spread of disease. The findings of this study also provide the foundation for understanding the evolution and genetic diversity of SARS-CoV-2.

## Data Availability

The spike gene sequence has been uploaded to GenBank under the following accession numbers: MT415366, MT415367, MT415368, MT415369, MT415370, MT415371, MT415372, MT415373, MT415374, MT415375, MT415376, MT415377.

## Abbreviations

COVID-19: Corona Virus Disease 2019
SARS-CoV-2: Severe acute respiratory syndrome coronavirus 2
CoV: Coronavirus
ICTV: International Committee on Taxonomy of Viruses
MERS-CoV: Middle East respiratory syndrome coronavirus
ARDS: Acute respiratory distress syndrome
WHO: World Health Organization
RBD: Receptor binding domain
NTD: N-terminal domain
CTD: C-terminal domain
ORF: Open reading frame
ACE2: Angiotensin-converting enzyme 2
RT-PCR: Reverse transcription polymerase chain reaction
rRT-PCR: Real-time reverse transcription polymerase chain reaction.
